# Evaluation of MODIS combined DT and DB AOD retrievals and their association with meteorological variables over Qena, Egypt

**DOI:** 10.1007/s10661-023-11118-8

**Published:** 2023-03-18

**Authors:** Mohamed Elshora

**Affiliations:** Department of Public Works Engineering, Faculty of Engineering, Tanta University, 31511 Tanta, Egypt

**Keywords:** MODIS, Aerosol optical depth (AOD), Dark target (DT), Deep blue (DB), AERONET, Meteorological variables, Regression

## Abstract

The purpose of this study is to validate the daily Terra-MODIS level 2 combined dark target (DT) and deep blue (DB) aerosol optical depth (AOD) retrievals with a spatial resolution of 10 km against the ground-based AERONET AOD data to be used in evaluating the air pollution and impact of meteorological variables over Qena, Egypt, in 2019. The regression analysis demonstrated an accepted agreement between the MODIS and AERONET AOD data with a correlation coefficient (*R*) of 0.7118 and 74.22% of the collocated points fall within the expected error (EE) limits. Quality flag filtering and spatial and temporal collocation were found to have a significant impact on the regression results. Quality flag filtering increased *R* by 0.2091 and % within EE by 17.97, spatial collocation increased *R* by 0.0143 and % within EE by 1.13, and temporal collocation increased *R* by 0.0089 and % within EE by 4.43. By validating the MODIS AOD data seasonally and analyzing the temporal distribution of the seasonal AOD data to show the retrieval accuracy variations between seasons, it was found that the MODIS AOD observations overestimated the AERONET AOD values in all seasons, and this may be because of underestimating the surface reflectance. Perhaps the main reason for the highest overestimation in summer and autumn is the transportation of aerosols from other regions, which changes the aerosol model in Qena, making accurate aerosol-type assumptions more difficult. Therefore, this study recommends necessary improvements regarding the aerosol model selection and the surface reflectance calculations. Temperature and relative humidity were found to have a strong negative relationship with a correlation of − 0.735, and both have a moderate association with AOD with a correlation of 0.451 and − 0.356, respectively. Because Qena is not a rainy city, precipitation was found to have no correlation with the other variables.

## Introduction

Aerosols are tiny liquid or solid particles suspended in the air (Lipponen et al., 2018). Different sources produce aerosols, some of which are natural, such as mineral dust and sea spray, and some are human activities, such as smoke from fires and car and factory emissions. Aerosols have a significant impact on human health, climate, agriculture, hydrological cycle, ecosystems, radiative balance, and cloud microphysical properties (Ali & Assiri, 2019). Therefore, spatial and temporal evaluations of aerosols from local to global scales are necessary. Also, an accurate description of the aerosol loading is required for air quality applications (Gouda et al., 2022; Gui et al., 2021). The air pollution in different regions differs in terms of the primary sources of pollutants, meteorological conditions, and geographical attributes (Barzeghar et al., 2022; Tariq et al., 2021). It was reported that aerosol concentrations and distribution are significantly influenced by meteorological variables (Al-Hamdan et al., 2019; Kayes et al., 2019).

Aerosol optical depth (AOD) is an essential product that determines the aerosol density in the atmosphere. While the ground-based networks provide accurate measurements of aerosol properties at high temporal and spectral resolutions, their spatial coverage is still limited and there is a lack of AOD data in many regions, such as remote and rural places (Chen et al., 2021). Satellite sensors overcome this spatial barrier by providing regular AOD measurements at low to high spatial resolutions (Kim et al., 2021). Various agencies have launched high-resolution satellites containing a range of advanced aerosol-sensing equipment to monitor the atmosphere (Elshora, 2021, 2022). The most popular sensors are Moderate Resolution Imaging Spectroradiometer, Visible Infrared Imaging Radiometer Suite, Advanced Very High-Resolution Radiometer, Multi-angle Imaging SpectroRadiometer, Sea-viewing Wide Field-of-view Sensor, Total Ozone Mapping Spectrometer, Along-Track Scanning Radiometer, Polarization and Directionality of the Earth’s Reflectances, Ozone Monitoring Instrument, and Medium Resolution Imaging Spectrometer (Bilal & Nichol, 2015).

MODIS instruments were installed on Terra satellite in 1999 and on Aqua satellite in 2002. They offer regular measurements of AOD in 36 spectral channels, 1 to 2 days temporal resolution, and spatial resolutions of 250 m, 500 m, and 1000 m (Wei et al., 2019a, b, c). The MODIS AOD data are based on the dark target (DT) (Kaufman et al., 1997) and deep blue (DB) (Hsu et al., 2013) algorithms. While the DT algorithm has high retrieval accuracy over dark areas, the DB algorithm retrieves AOD data over high reflectance surfaces (Ali et al., 2022; Wang et al., 2017a). Another MODIS AOD algorithm is the combined DT and DB, with which its AOD measurements are produced based on the Normalized Difference Vegetation Index (NDVI) as follows: (1) If the NDVI is more than 0.3, the DT retrievals are considered; (2) if the NDVI is less than 0.2, the DB retrievals are considered; (3) if the NDVI is more than 0.2 and less than 0.3, the average of the retrievals from the DT and DB or the one that passes the required quality assurance (QA = 3 for DT and QA ≥ 2 for DB) is considered (Wei et al., 2019a, b, c).

MODIS AOD products are used to estimate and map fine particulate matter concentrations from local to global scales. As a result, it is necessary to assess the quality of MODIS AOD observations using accurate AOD measurements from the Aerosol Robotic Network (AERONET) in order to produce accurate and reliable air quality maps (Bilal et al., 2016). The correlation coefficient (*R*), the root-mean-square error (RMSE), the EE, and the relative mean bias (RMB) are the parameters that assess the relationship between the MODIS and AERONET AOD data. To verify that the AOD identified by the MODIS sensor from space and the AOD measured by the AERONET on the ground are acquired at the same time and place, the spatial and temporal collocation process is required (Osgouei et al., 2022). While the spatial collocation is achieved by averaging the MODIS AOD retrievals within a 5 × 5-pixel sampling window centered on the AERONET location, the temporal collocation is achieved by averaging the AERONET AOD data within ± 30 min of each MODIS AOD retrieval time. Only the high-quality MODIS AOD retrievals with a quality flag (QF) = 3 will be used as they have the lowest errors (Deep et al., 2021; Wang et al., 2020).

Despite the several sources of aerosol in Qena, Egypt, there is a gap in the literature on studying its air pollution and the impact of meteorological variables. Therefore, this study aims to validate the Terra-MODIS combined DT and DB AOD data at a spatial resolution of 10 km against the ground-based AERONET AOD measurements to be used in studying the air pollution over Qena in 2019. This study presents the impact of quality flag filtering and spatial and temporal collocation, which have not been sufficiently studied in the literature. This study also proposes validating the MODIS AOD data seasonally and analyzing the temporal distribution of the seasonal AOD data to show the retrieval accuracy variations between seasons. Furthermore, this study investigates the impact of meteorological variables on AOD concentrations. The daily variations of temperature (T in °C), relative humidity (RH in %), and precipitation (P in mm/day) are analyzed to evaluate their associations with AOD in addition to the associations between each other.

## Study area and datasets

### Study area

**Fig. 1:**
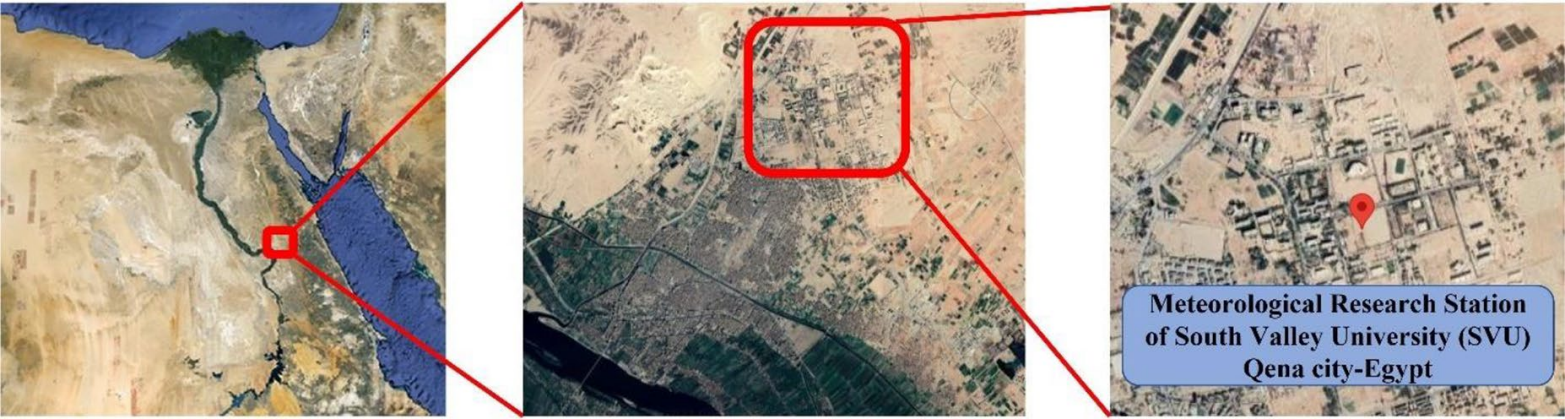
The study area

As shown in Fig. 1, Qena City is in the southern part of Egypt, on the east bank of the Nile. The study site is located at the meteorological research station of South Valley University (SVU) at Qena City (26.200° N, 32.747° E, and 75.7 m. elevation). The station is in a semi-desert area about 6 km northeast of Qena City, with low agricultural areas around it. The primary causes of pollution in Qena City include sand from the surrounding hills, vehicle emissions, and industrial pollutants from various sources such as aluminum, sugar, and cement factories.

### AERONET data

The AERONET is a network of Sun photometers on the ground that provides a long-term, continuous, and easily accessible public database of aerosol optical and radiative properties for aerosol characterization and validation of satellite retrievals. Version 3 algorithm processing includes three levels of quality: (1) data at level 1.0 has not been cloud-screened or calibrated; (2) data at level 1.5 has been cloud-screened but not calibrated or quality-assured; and (3) data at level 2.0 has been cloud-screened, calibrated, and quality-assured. As AERONET AOD data has low uncertainty (0.01–0.02) and high temporal resolution (every 15 min), it is used for the validation of satellite AOD products (Filonchyk et al., 2020). The highest quality level 2.0 data from Qena-SVU station in 2019 will be used in this research.

### MODIS data

The Moderate Resolution Imaging Spectroradiometer (MODIS) is a primary instrument on the Terra and Aqua satellites. Terra crosses the equator in the morning from north to south, while Aqua crosses the equator in the afternoon from south to north. Every 1–2 days, Terra and Aqua MODIS observe the whole Earth’s surface and collect the data in the available 36 spectral bands or sets of them. The quality of MODIS AOD data is regularly improved by updating cloud screening, surface reflectance estimation, the dynamic aerosol model, and gas and Rayleigh corrections. This study will use the daily Terra MODIS level-2 data (MOD04_L2) acquired in 2019 based on the combined DT and DB algorithm, with a temporal resolution of 5 min and a spatial resolution of 10 km.

### Meteorological data

The meteorological parameters have a strong impact on the AOD concentrations and distribution. The 24-h daily mean temperature (T in °C), relative humidity (RH in %), and precipitation (P in mm/day) were acquired for 2019 over Qena. The temperature and relative humidity data was obtained from the Atmospheric Infrared Sounder (AIRS) level 3 daily gridded product (AIRS3STD V7.0). AIRS is a grating spectrometer on the Aqua satellite that collects infrared radiation from Earth’s surface and atmosphere with a spatial resolution of 1° and provides measurements of temperature, water vapor, trace gases, and surface and cloud characteristics. The precipitation data was obtained from the Global Precipitation Climatology Project (GPCP) datasets (GPCPDAY v3.2), which offers daily precipitation analysis based on surface and satellite measurements with a spatial resolution of 0.5°.

## Methodology

To evaluate and validate MODIS AOD retrievals, they should be fitted in space and time with AERONET AOD measurements. The MODIS sensor offers spatial coverage of a region from space, while the AERONET delivers measurements at a specific location on the ground. Because of the variations in the atmosphere from one location to another, as well as the movement of the atmosphere through time, the spatial and temporal collocation process is required to ensure that the AOD detected by the MODIS sensor from space and the AOD measured by the AERONET on the ground are acquired at the same time and place (Ichoku et al., 2002). The spatial collocation is performed by averaging at least two pixels of MODIS AOD retrievals within a 5 × 5-pixel sampling window centered on the AERONET location, while the temporal collocation is performed by averaging at least two AERONET AOD values within ± 30 min of each MODIS AOD retrieval time (Wang et al., 2017b). Since the MODIS AOD data wavelength is 550 nm and the AERONET AOD data wavelength is 500 nm, the empirical approach, standard angstrom exponent (α), is used to interpolate the wavelength of the AERONET AOD measurements from 500 to 550 nm (Falah et al., 2021), according to Eq. (1).

1 $${\mathrm{AOD}}_{550\mathrm{ nm}}= {\mathrm{AOD}}_{500\mathrm{ nm}} \times {(\frac{550}{500})}^{-\alpha }$$

The MODIS algorithms use a protocol to assess the AOD retrieval quality, which is known as the quality assurance (QA). The QA protocol includes a group of tests to check that specific conditions are met during the retrieval and, as a result, provides a degree of confidence for the entire retrieving process. The degree of confidence ranges from 3 (high confidence) to 0 (no confidence) (Levy et al., 2010; Remer et al., 2005). Only the MODIS combined DT and DB AOD retrievals with quality flag (QF) = 3 are recommended to be used since they are characterized by the lowest absolute uncertainty and the highest proportions inside the EE.

The relationship between the MODIS AOD retrievals and their collocated AERONET AOD measurements was evaluated using a linear regression analysis according to Eq. (2). The regression line’s slope and intercept were estimated to determine the suitability of the aerosol model and surface reflectance assumptions, which are responsible for the MODIS AOD retrieval quality (Ali et al., 2017). The slope describes the error of the improper selection of the aerosol model, while the intercept describes the unsuitable surface reflectance assumptions. The ideal values of the slope and intercept are 1 and 0, respectively, which indicates that the MODIS AOD retrievals have the best quality. The positive intercept indicates a surface reflectance underestimation, while the negative intercept indicates a surface reflectance overestimation.

The correlation coefficient (*R*) and the RMSE were calculated according to Eqs. (3) and (4), respectively, to assess the validation quality and indicate how accurate the MODIS AOD observations is against the AERONET AOD measurements. While the *R* measures the agreement between the MODIS and AERONET AOD data, the RMSE measures the differences between them. The higher *R* and lower RMSE suggest that there is more agreement. The EE is calculated according to Eq. (5) to assess the AOD retrieval uncertainty. The more AOD retrievals that fall inside the EE (%within), the higher the confidence of the retrieval algorithm. The RMB, which indicates the mean bias of the MODIS and AERONET AOD data, is calculated according to Eq. (6). RMB > 1 represents the average overestimation of the retrievals, and RMB < 1 represents the average underestimation of the retrievals. To test the statistical significance of the regression, the probability value (*P*-value) is calculated. The *P*-value is a statistical measurement used to check the validity of a hypothesis against actual data (Lalitaporn & Mekaumnuaychai, 2020). A *P*-value of 0.05 or lower indicates that the relationship is statistically significant.

2 $$\mathrm{MODIS}\;\mathrm{AOD}=\mathrm{slope}\;\times\;\mathrm{AERONET}\;\mathrm{AOD}\;+\;\mathrm{intercept}$$

3 $$R=\frac{\sum_{i=1}^n\left({\mathrm{AERONET}\;\mathrm{AOD}}_i-\mathrm{AERONET}\;{\mathrm{AOD}}_m\right)\left(\mathrm{MODIS}\;{\mathrm{AOD}}_i-\mathrm{MODIS}\;{\mathrm{AOD}}_m\right)}{\sqrt{\sum_{i=1}^n\left(\mathrm{AERONET}\;{\mathrm{AOD}}_i-\mathrm{AERONET}\;{\mathrm{AOD}}_m\right)^2\sum_{i=1}^n\left(\mathrm{MODIS}\;{\mathrm{AOD}}_i-\mathrm{MODIS}\;{\mathrm{AOD}}_m\right)^2}}$$

4 $$\mathrm{RMSE}=\sqrt{\frac1n{\textstyle\sum_{i=1\;}^n}{(\mathrm{MODIS}\;{\mathrm{AOD}}_i-\mathrm{AERONET}\;{\mathrm{AOD}}_i)}^2}$$

5 $$\mathrm{EE}=\pm(0.05+0.20\;\mathrm{AERONET}\;\mathrm{AOD})$$

6 $$\mathrm{RMB}=\frac{\mathrm{MODIS}\;{\mathrm{AOD}}_m}{\mathrm{AERONET}\;{\mathrm{AOD}}_m}$$

The impact of quality flag filtering, spatial collocation, and temporal collocation on the results of the validation of MODIS AOD data was analyzed by comparing the results of (1) using only high-quality MODIS AOD data and using all AOD data; (2) using a 5 × 5-pixel typical window (± 25 km) and using a 3 × 3-pixel typical window (± 15 km); and (3) using the average of only AERONET AOD values within ± 30 min of each MODIS AOD retrieval time and using the average of all AERONET AOD values over the day. The MODIS AOD retrievals in each season were validated separately, and the temporal distribution of the seasonal means of both MODIS and AERONET AOD observations was analyzed to show the seasonal variations in the quality of AOD retrieval. Because of the strong impact of the meteorological parameters on the AOD concentrations and distribution, the daily variations of AOD (550 nm), temperature (T in °C), relative humidity (RH in %), and precipitation (P in mm/day) were analyzed over Qena in 2019. The statistical parameters: minimum, maximum, mean, standard deviation, median, and 95th percentile, were calculated for analyzing the AOD and the meteorological variables. The association between the AOD and the meteorological parameters was assessed by the linear regression analysis. In addition, the correlation coefficients between any two of the four variables were calculated.

## Results

### Validation of MODIS AOD retrievals

**Fig. 2:**
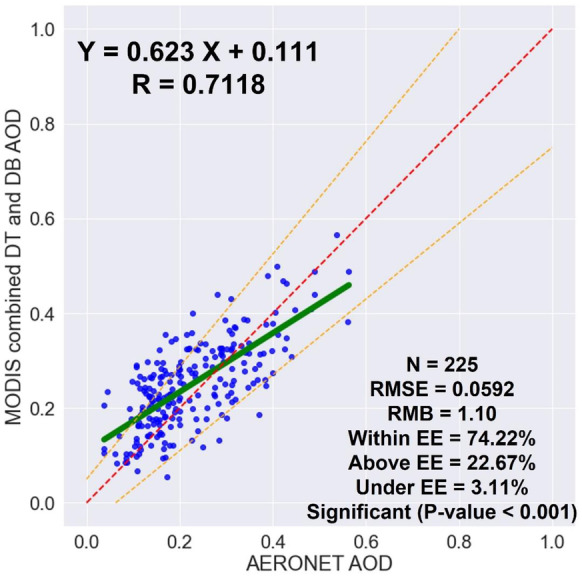
The validation of MODIS AOD retrievals by using the AERONET AOD measurements, the green solid line indicates the regression line, the red dashed line indicates the line of slope 1:1, and the orange dashed lines indicate the EE range

By using the high-quality MODIS AOD retrievals within a 5 × 5-pixel sampling window centered on the AERONET location and the AERONET AOD values within ± 30 min of each MODIS AOD retrieval, 225 collocated points participated in the comparison. A linear regression analysis was used to assess the relationship between the MODIS and AERONET AOD data, as shown in Fig. 2. The regression line has a slope of 0.623 and an intercept of 0.111, which indicates an improper aerosol model and that the surface reflectance was partially underestimated, respectively. Overall, there is an accepted agreement between the MODIS and AERONET AOD data with *R* = 0.7118 and RMSE = 0.0592. The MODIS AOD retrievals were more regularly scattered on both sides of the 1:1 line, and 74.22% of the collocated points fall inside the EE limits. With an RMB of 1.10, the MODIS combined DT the DB AOD algorithm shows an average overestimation of 10%, where 22.67% of the collocated points fall above the EE and 3.11% fall below the EE. Generally, the validation analysis shows that the aerosol situation over Qena can be well represented by the MODIS combined DT and DB AOD product. It is important to analyze (1) the impact of quality flag filtering by conducting a comparison between the results of using only high-quality MODIS AOD data and using all AOD data, (2) the impact of spatial collocation by conducting a comparison between the results of using a 5 × 5-pixel typical window (± 25 km) and using a 3 × 3-pixel typical window (± 15 km), and (3) the impact of temporal collocation by conducting a comparison between the results of using the average of only AERONET AOD values within ± 30 min of each MODIS AOD retrieval time and using the average of all AERONET AOD values over the day.

#### Quality flag filtering

**Fig. 3:**
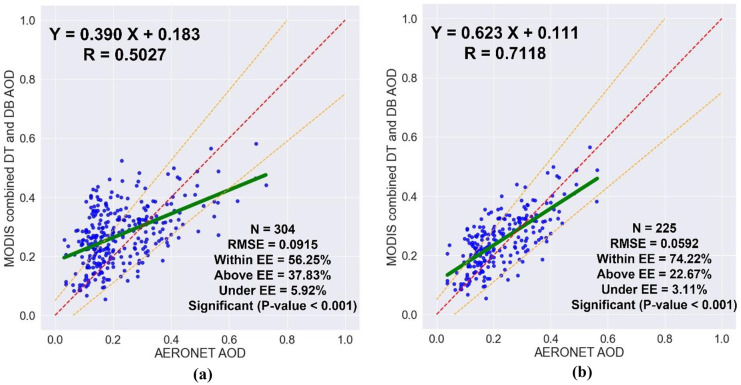
The impact of quality flag filtering on the validation of MODIS AOD retrievals; **a** all retrievals, and **b** high-quality only

Only the MODIS AOD retrievals with QF ≥ 3 are recommended to be used since they are characterized by the lowest absolute uncertainty and the highest proportions inside the EE. To assess the impact of MODIS AOD retrieval quality on the relationship between the MODIS AOD retrievals and their collocated AERONET AOD measurements, a comparison was performed between the results of averaging all MODIS AOD data and averaging only the high-quality MODIS AOD data, as shown in Table 1 and Fig. 3. A 5 × 5-pixel sampling window was utilized to calculate the average of the MODIS AOD retrievals, and the AERONET AOD values within ± 30 min of each MODIS AOD retrieval time were used. The number of collocations generated by using the high-quality MODIS AOD retrievals was less than that generated by using all retrievals by 26%. By using only high-quality MODIS AOD retrievals instead of using all retrievals, the correlation coefficient, and the proportion of collocations within the EE increased from 0.5027 to 0.7118 and from 56.25 to 74.22, respectively, and the root-mean-square error decreased from 0.0915 to 0.0592. Therefore, using the high-quality MODIS AOD retrievals is recommended.

**Table 1:** The impact of quality flag filtering on the validation of MODIS AOD retrievals

**MODIS AOD retrievals within a 5 × 5-pixel window and AERONET AOD measurements within ± 30 min**				
**Dataset**	*N*	*R*	RMSE	%Within
**All retrievals**	304	0.5027	0.0915	56.25
**High-quality only**	225	0.7118	0.0592	74.22

#### Spatial collocation

**Fig. 4:**
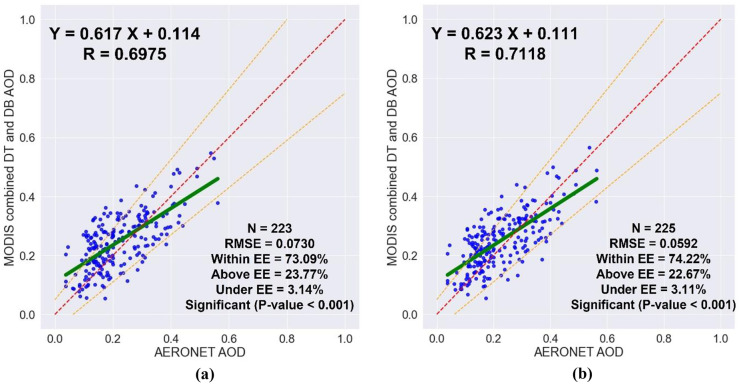
The impact of the sampling window size on the validation of MODIS AOD retrievals; **a** 3 × 3-pixel typical window (± 15 km), and **b** 5 × 5-pixel typical window (± 25 km)

A sampling window centered on the AERONET location is used to perform the spatial collocation and the average of all pixels of MODIS AOD retrievals within the window is calculated. To assess the impact of the sampling window size on the relationship between the MODIS AOD retrievals and their collocated AERONET AOD measurements, the results of a 3 × 3-pixel typical window (± 15 km) and a 5 × 5-pixel typical window (± 25 km) were evaluated and compared, as shown in Table 2 and Fig. 4. Only the high-quality MODIS AOD retrievals, with QF ≥ 3, within the window were averaged and compared to the AERONET AOD values within ± 30 min of each MODIS AOD retrieval time. The number of collocations generated by using a larger 5 × 5-pixel window (± 25 km) was higher than that generated by using a smaller 3 × 3-pixel window (± 15 km) by 1%. By increasing the size of the sampling window from 3 × 3 pixels to 5 × 5 pixels, the correlation coefficient, and the proportion of collocations within the EE increased from 0.6975 to 0.7118 and from 73.09 to 74.22, respectively, and the root-mean-square error decreased from 0.0730 to 0.0592. Therefore, the 5 × 5-pixel sampling window that uses the MODIS AOD retrievals within ± 25 km is recommended.

**Table 2:** The impact of the sampling window size on the validation of MODIS AOD retrievals

**High-quality MODIS AOD retrievals and AERONET AOD measurements within ± 30 min**				
**Window**	*N*	*R*	RMSE	%Within
**3×3**	223	0.6975	0.0730	73.09
**5×5**	225	0.7118	0.0592	74.22

#### Temporal collocation

**Fig. 5:**
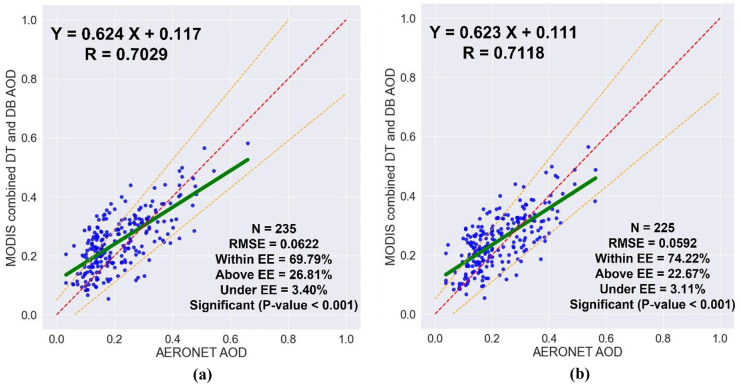
The impact of temporal collocation on the validation of MODIS AOD retrievals; **a** daily average, and **b** within ± 30 min

The average of AERONET AOD values within ± 30 min of each MODIS AOD retrieval time is calculated to perform the temporal collocation. To assess the importance of the temporal collocation on the relationship between the MODIS AOD retrievals and their collocated AERONET AOD measurements, a comparison was performed between the results of averaging all AERONET AOD data over the day and averaging only AERONET AOD data within ± 30 min of each MODIS AOD retrieval time, as shown in Table 3 and Fig. 5. Only the high-quality MODIS AOD retrievals, with QF ≥ 3, within a 5 × 5-pixel sampling window were averaged and compared to the AERONET AOD measurements. The number of collocations generated by using the AERONET AOD measurements within ± 30 min was less than that generated by using all measurements over the day by 4%. By using only AERONET AOD data within ± 30 min instead of using all the AERONET AOD data over the day, the correlation coefficient, and the proportion of collocations within the EE increased from 0.7029 to 0.7118 and from 69.79 to 74.22, respectively, and the root-mean-square error decreased from 0.0622 to 0.0592. Therefore, using the average of only AERONET AOD values within ± 30 min of each MODIS AOD retrieval time is recommended.

**Table 3:** The impact of temporal collocation on the validation of MODIS AOD retrievals

**High-quality MODIS AOD retrievals within a 5 × 5-pixel window and AERONET AOD measurements**				
**Dataset**	*N*	*R*	RMSE	%Within
**AERONET AOD (daily average)**	235	0.7029	0.0622	69.79
**AERONET AOD (within ± 30 min)**	225	0.7118	0.0592	74.22

### Seasonal validation of MODIS AOD retrievals

**Fig. 6:**
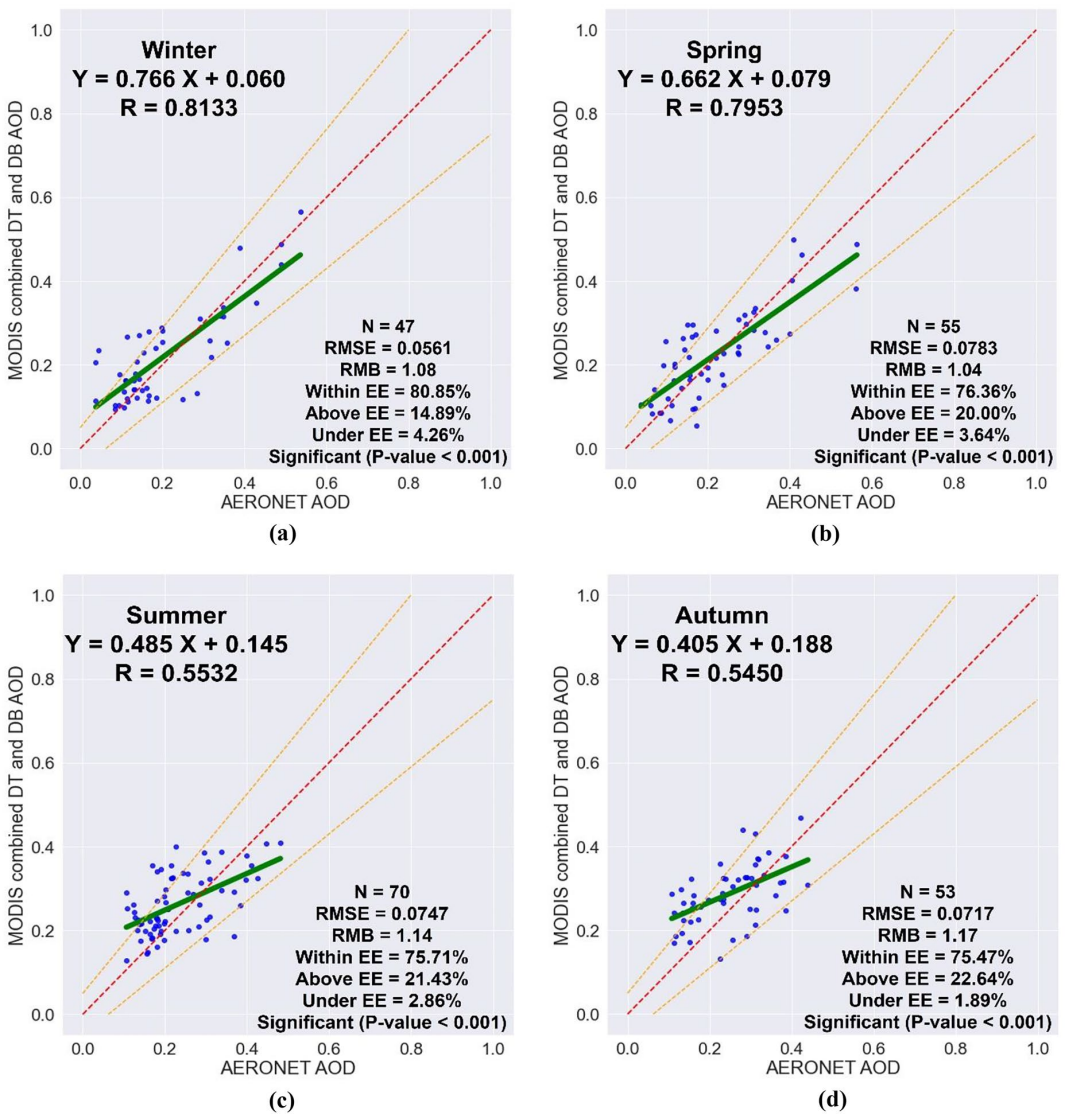
The seasonal validation of MODIS AOD retrievals by using the AERONET AOD measurements, **a** winter, **b** spring, **c** summer, and **d** autumn

The seasonal comparisons between the MODIS and AERONET AOD data are shown in Fig. 6. Seasonal variations in slope values have been discovered. The best slope was found in winter with a value of 0.766, followed by 0.662 in spring and 0.485 in summer, while the worst slope was found in autumn with a value of 0.405. This indicates that improvements are required in aerosol model selection. The intercept values were also different for all seasons. The winter showed the best intercept with a value of 0.060, which indicates a low underestimation of the surface reflectance, followed by 0.079 in spring and 0.145 in summer, while the autumn showed the worst intercept with a value of 0.188, which indicates a high underestimation of the surface reflectance. The *R* values varied in different seasons; the winter and spring showed a high correlation with values of 0.8133 and 0.7953, respectively, while the summer and autumn showed a poor correlation with values of 0.5532 and 0.5450, respectively. The RMSE values for all seasons were different. The lowest RMSE was found in winter with a value of 0.0561, followed by 0.0717, 0.0747, and 0.0783 for autumn, summer, and spring. The highest percentage of collocated points that fall within the EE limits was found in winter with a value of 80.85%, followed by 76.36% in spring, 75.71% in summer, and 75.47% in autumn. The RMB values were 1.08, 1.04, 1.14, and 1.17 for winter, spring, summer, and autumn, respectively. The RMB values indicate the average overestimation of the MODIS combined DT and DB algorithm in retrieving AOD, where 14.89% of the collocated points fall above the EE for winter, 20.00% for spring, 21.43% for summer, and 22.64% for autumn.

### Seasonal distribution of MODIS and AERONET AOD observations

**Fig. 7:**
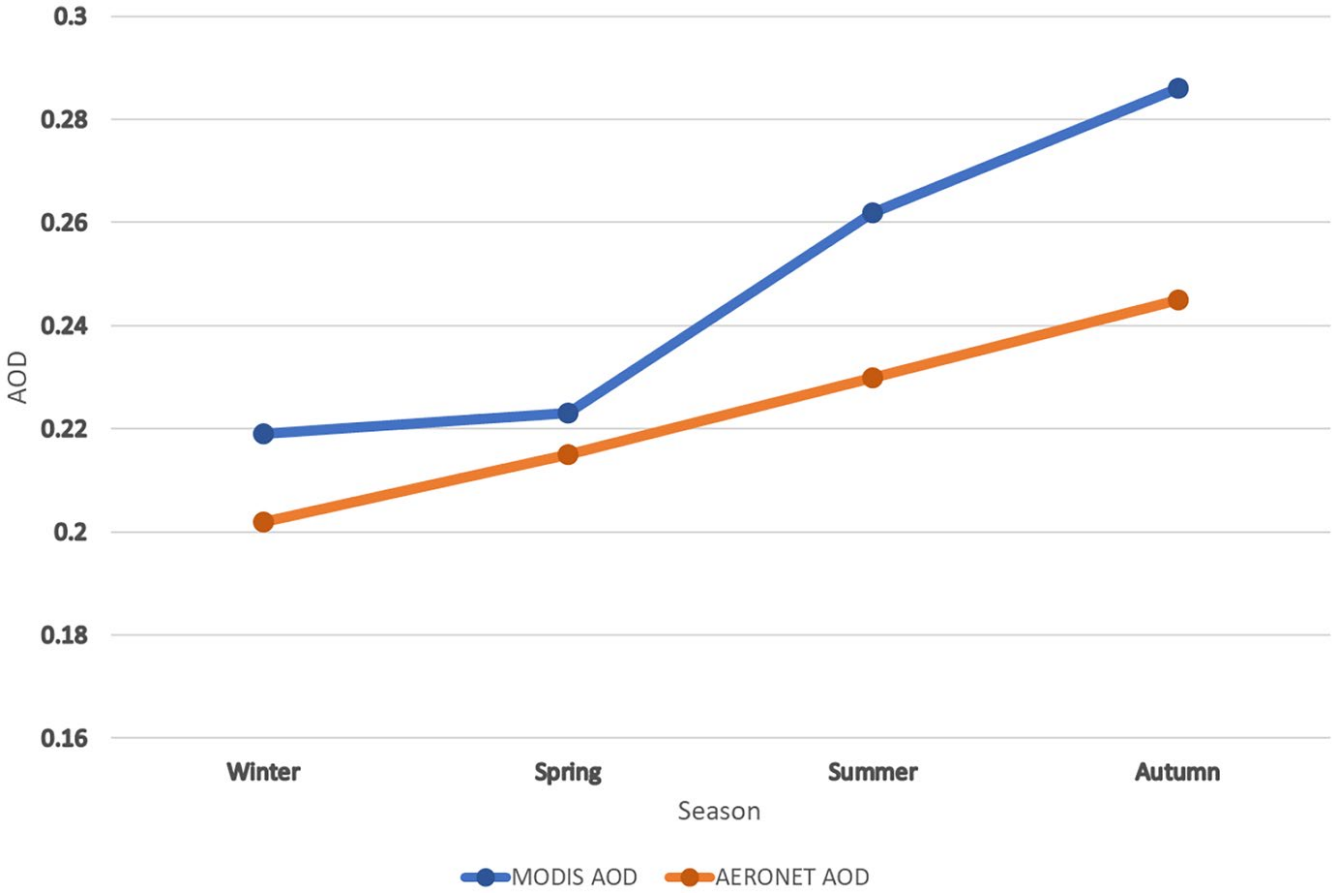
The seasonal distribution of MODIS and AERONET AOD observations

The seasonally means of both MODIS and AERONET AOD data were calculated and temporally analyzed for all seasons during 2019. According to Fig. 7, the seasonal MODIS AOD data follows the same trend of the seasonal AERONET AOD data. The winter shows the lowest AOD values with 0.219 for MODIS and 0.202 for AERONET, while the autumn shows the highest AOD values with 0.286 for MODIS and 0.245 for AERONET. All AOD values are less than 0.3 and this indicates a moderate air quality index. The MODIS AOD data are always overestimated for all seasons compared to the AERONET AOD data. The low overestimation was found in winter and spring, while the high overestimation was found in summer and autumn. These outcomes strongly recommend using the MODIS combined DT and DB aerosol product to represent the air quality situation over Qena.

### Association between AOD and meteorological parameters

**Fig. 8:**
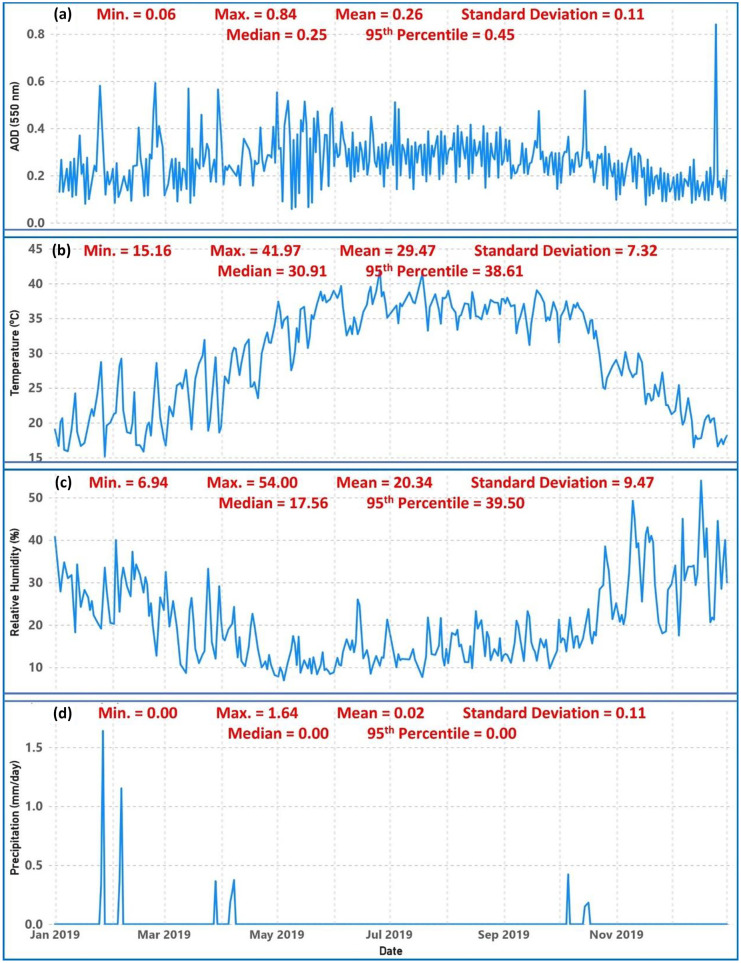
Temporal variation of **a** AOD, **b** temperature, **c** relative humidity, and **d** precipitation over Qena during 2019

The meteorological parameters, such as temperature, relative humidity, and precipitation, have a significant impact on the AOD concentrations and distribution. Figure 8a shows the daily variation of AOD (550 nm) over Qena in 2019. The annual mean of the daily AOD values is 0.26 ± 0.11. It is noted from the figure that the maximum value of AOD was measured in December with a value of 0.84, while the minimum value of AOD was measured in May with a value of 0.06. Figure 8b shows the daily variation of temperature (°C) over Qena in 2019. The annual mean of the daily temperature values is 29.47 ± 7.32 (°C). It is noted from the figure that the daily temperature fluctuation was greatest in June–September and lowest in December–February. While the maximum temperature value was measured in June with a value of 41.97 (°C), the minimum temperature value was measured in January with a value of 15.16 (°C). Figure 8c shows the daily variation of relative humidity (%) over Qena in 2019. The annual mean of the daily relative humidity values is 20.34 ± 9.47 (%). It is noted from the figure that the daily fluctuation of relative humidity follows an inverse pattern to that of temperature, where it was greatest in November–February and lowest in May–September. While the maximum relative humidity value was measured in December with a value of 54.00 (%), the minimum relative humidity value was measured in May with a value of 6.94 (%). Figure 8d shows the daily variation of precipitation (mm/day) over Qena in 2019. The annual mean of the daily precipitation values is 0.02 ± 0.11 (mm/day). It is noted from the figure that the number of rainy days in 2019 was very low (12 days) and the annual total precipitation was 5.62 (mm). The maximum precipitation values were measured in January and February, with values of 1.64 and 1.15 (mm/day), respectively.

**Fig. 9:**
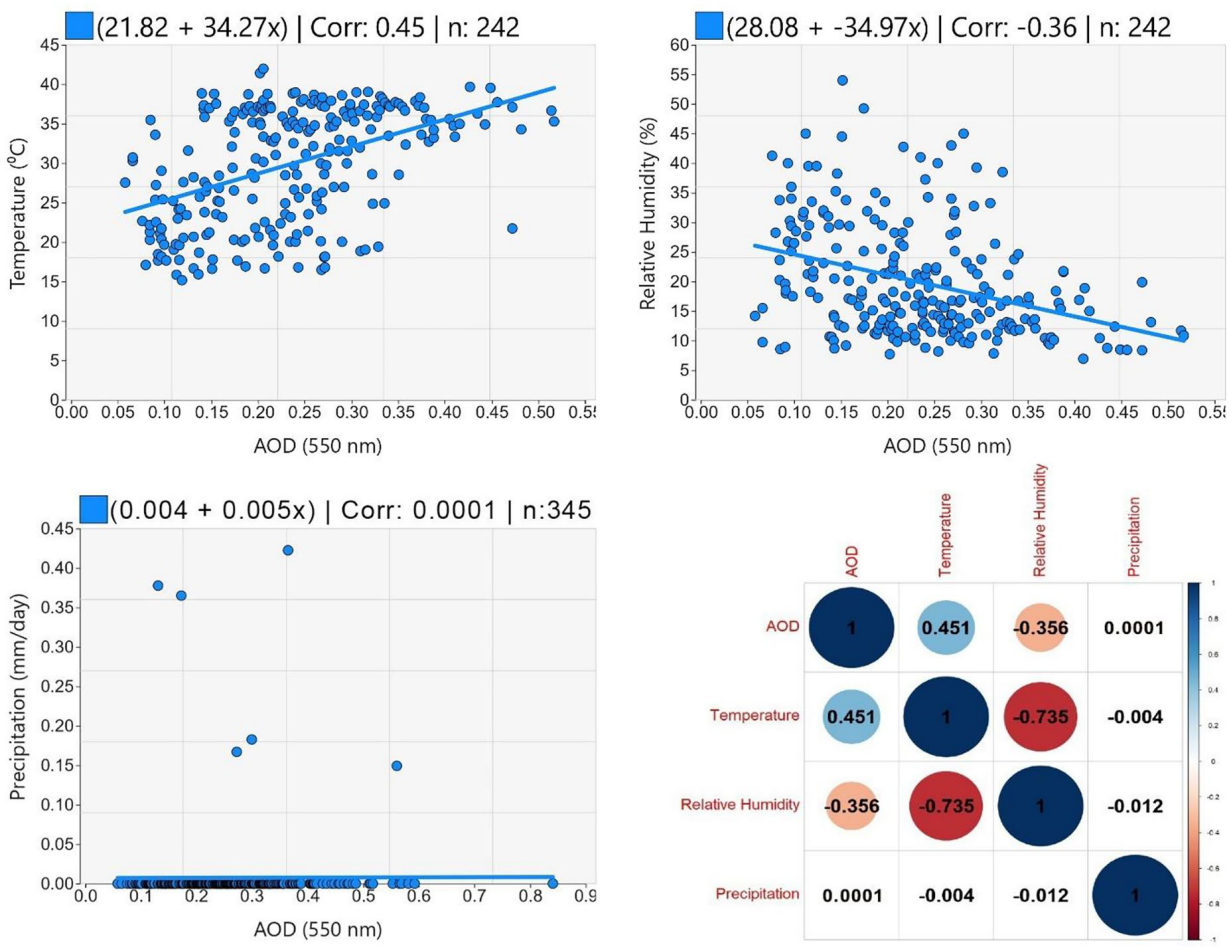
Association between AOD and meteorological parameters over Qena during 2019

A linear regression analysis was used to assess the relationship between the AOD and the meteorological parameters over Qena during 2019, as shown in Fig. 9. The regression between AOD and temperature, relative humidity, and precipitation used 242, 242, and 345 coincident observations, respectively. The resultant regression equations have an intercept of 21.82 (°C), 28.08 (%), and 0.004 (mm/day) and a slope of 34.27, 34.97, and 0.005 for temperature, relative humidity, and precipitation, respectively, against AOD. The correlations between AOD and temperature, relative humidity, and precipitation are a positive correlation (*R* = 0.45), a negative correlation (*R* =  − 0.36), and a positive correlation (*R* = 0.0001), respectively. The correlation coefficients between any two of the four variables are shown in Fig. 9. It is evident that temperature and relative humidity have a strong negative correlation, which indicates that the days of high temperature have low relative humidity and vice versa. Both temperature and relative humidity have moderate correlations with AOD. Due to the low precipitation over Qena, it has weak correlations with the other variables.

## Discussion

Qena is one of the largest cities in Egypt that has many sources of air pollution, such as sand from the surrounding hills, vehicle emissions, and industrial pollutants from various sources such as aluminum, sugar, and cement factories. However, there is limited literature on studying the air pollution over Qena and the impact of the meteorological variables. The MODIS combined DT and DB AOD product well represented the aerosol situation over Qena with an accepted agreement with the AERONET AOD data (*R* = 0.7118, and % within EE = 74.22). Many publications validated the MODIS AOD products and demonstrated their highest quality. Wei et al. (2019a, b, c) evaluated the Terra and Aqua MODIS DT, DB, and combined DT and DB AOD products over land during 2003–2017 against the AERONET AOD measurements from 431 sites around the world and demonstrated that the MODIS AOD retrievals are well correlated with the AERONET AOD measurements globally and the DTB AOD product outperforms the other products in most regions (*R* = 0.843 to 0.844, and % within EE = 67.01 to 73.82). Bilal et al. (2018) validated the MODIS DT and DB AOD product against the AERONET AOD measurements from 68 global sites located over various vegetated surfaces from 2004 to 2014 and demonstrated a strong agreement (*R* = 0.81 to 0.91, and % within EE = 61.00 to 75.00). Tian et al. (2019) assessed the accuracy of Terra MODIS DT, DB, and combined DT and DB AOD products for the years 2000–2016 against the AERONET AOD measurements from 23 sites situated in heavy aerosol loading areas and demonstrated a high accuracy for the combined DT and DB AOD products (*R* = 0.841, and % within EE = 53.1). There are many other publications that used the MODIS AOD products to evaluate the air pollution over different regions, such as Bilal et al. (2021), who provided a comprehensive evaluation of the air quality over Pakistan during 2003–2020, and Nichol et al. (2020), who studied the air pollution scenario during COVID-19 over China.

One of the main contributions of this study is analyzing the importance of quality flag filtering and spatial and temporal collocation. Using only high-quality MODIS AOD retrievals improved *R* by 0.2091 and % within EE by 17.97. Using only AERONET AOD data within ± 30 min of each MODIS AOD retrieval time improved *R* by 0.0089 and % within EE by 4.43. Increasing the size of the sampling window from 3 × 3 pixels to 5 × 5 pixels improved *R* by 0.0143 and % within EE by 1.13. There are not many publications that discuss the impact of quality flag filtering and spatial and temporal collocation. Osgouei et al. (2022) presented one of these few publications, which provided an evaluation of the AOD products over regions in the Eastern Mediterranean and the Black Sea between 2014 and 2018, concluding that using only high-quality MODIS AOD retrievals increases *R* by a value between 0 and 0.14, and % within EE by a value between 3.21 and 15.68, and using only AERONET AOD data within ± 30 min of each MODIS AOD retrieval time increases *R* by a value between 0.02 and 0.11, and % within EE by a value between 1.61 and 2.68. In addition, he used a typical window of 5 × 5 pixels for AOD products with different spatial resolutions and concluded that choosing a sampling window depends on the spatial resolution of the AOD products.

The MODIS AOD retrievals were validated seasonally and showed that the best retrieval accuracy was in winter, while the worst was in autumn. It is necessary to report that the seasonal accuracy of MODIS AOD retrievals varies in the different regions because of the different land use and meteorological conditions, and this was confirmed in the study presented by Su et al. (2021), who evaluated different AOD products from 2016 to 2020 over different sites in East China. The MODIS DT and DB AOD observations overestimated the AERONET AOD values for all seasons, and this may be because of the underestimation of the surface reflectance (Che et al., 2019). Furthermore, the highest overestimation in summer and autumn may be because of the transportation of aerosols from other regions, which leads to changing the aerosol model in Qena, making accurate aerosol-type assumptions more difficult (Chen et al., 2019).

The daily variations of the meteorological variables: temperature, relative humidity, and precipitation were analyzed over Qena in 2019 to study their impact on the AOD concentrations. The linear regression analysis was utilized to assess the association between the AOD and the meteorological parameters, which demonstrated that temperature has a moderate positive correlation with AOD, relative humidity has a moderate negative correlation, and precipitation has no correlation. Moreover, the correlation coefficients between any two of the four variables were calculated, which showed that temperature and relative humidity have a strong negative correlation, while precipitation has weak correlations with the other variables. Many publications have reported the impact of temperature and relative humidity on AOD, such as Kang et al. (2020) and Ali et al. (2020). Perhaps the low rainfall over Qena is the main reason for the non-existence of a relationship with the AOD. According to some publications, there is no clear relationship between precipitation and AOD (Ramachandran et al., 2012; Jin et al., 2005). However, some other publications reported that rainfall has an impact on AOD concentrations, such as Boiyo et al. (2018), Makokha et al. (2017), and Chowdhury et al. (2016), which demonstrated that rainfall reduces the emission of dust while soil moisture limits the formation of post-precipitation aerosols. It is evident that the impact of the meteorological parameters on AOD differs across different places because of the differences in meteorological conditions and aerosol types, sources, and transportation processes.

## Conclusion

In this research, the daily Terra-MODIS level 2 combined DT and DB AOD data (MOD04_L2) with a spatial resolution of 10 km was validated against the ground-based AERONET AOD data and used to study the air pollution over Qena, Egypt, in 2019. Because of the significant impact of quality flag filtering and spatial and temporal collocation, the regression analysis demonstrated an accepted agreement between the MODIS and AERONET AOD data with *R* = 0.7118 and % within EE = 74.22. Therefore, it is recommended to study the impact of quality flag filtering and spatial and temporal collocation globally in places with different land cover, meteorological conditions, and pollution sources. However, the MODIS AOD observations overestimated the AERONET AOD values in all seasons because of underestimating the surface reflectance. Moreover, the highest overestimation in summer and autumn may be because of the transportation of aerosols from other regions, which changes the aerosol model in Qena, making accurate aerosol-type assumptions more difficult. As a result, it is recommended to improve aerosol model selection and surface reflectance calculations. Additionally, wind speed and direction should be incorporated into the meteorological variables to be studied. Temperature and relative humidity have a strong negative correlation, and both have a moderate correlation with AOD. Because of the low precipitation over Qena, it has weak correlations with the other variables. The impact of meteorological parameters on AOD fluctuates across different places because of the disparities in meteorological conditions and aerosol types, sources, and transportation processes. As a future work for Qena, other criteria air pollutants such as PM_10_, PM_2.5_, SO_2_, NO_2_, CO, and O_3_ and their associations with meteorological variables will be analyzed. In addition, the relationship between columnar AOD and surface PM_2.5_ will be studied.

## Data Availability

MODIS data is freely available at Level-1 and Atmosphere Archive & Distribution System (LAADS), Distributed Active Archive Center (DAAC) (https://ladsweb.modaps.eosdis.nasa.gov/), AERONET data is freely available at the aerosol robotic network website (https://aeronet.gsfc.nasa.gov/), and meteorological data is freely available at the Giovanni website (https://giovanni.gsfc.nasa.gov/giovanni/).

## References

[CR1] Al-Hamdan, M., Crosson, W., Burrows, E., Coffield, S., Crane, B., & Barik, M. (2019). Development and validation of improved PM2.5 models for public health applications using remotely sensed aerosol and meteorological data. *Environmental Monitoring and Assessment,**191*, 328. 10.1007/s10661-019-7414-3 10.1007/s10661-019-7414-3 31254078

[CR2] Ali G, Bao Y, Ullah W, Ullah S, Guan Q, Liu X, Li L, Lei Y, Li G, Ma J (2020). Spatiotemporal trends of aerosols over urban regions in Pakistan and their possible links to meteorological parameters. Atmosphere.

[CR3] Ali, M. A., Bilal, M., Wang, Y., Qiu, Z., Nichol, J. E., Leeuw, G., Ke, S., Mhawish, A., Almazroui, M., Mazhar, U., Habtemicheal, B. A., & Islam, M. N. (2022). Evaluation and comparison of CMIP6 models and MERRA-2 reanalysis AOD against satellite observations from 2000 to 2014 over China. *Geoscience Frontiers,**13*(2), 101325. 10.1016/j.gsf.2021.101325

[CR4] Ali MA, Assiri M (2019). Analysis of AOD from MODIS-merged DT–DB products over the Arabian Peninsula. Earth Systems and Environment.

[CR5] Ali MA, Assiri M, Dambul R (2017). Seasonal aerosol optical depth (AOD) variability using satellite data and its comparison over Saudi Arabia for the period 2002–2013. Aerosol and Air Quality Research.

[CR6] Barzeghar, V., Hassanvand, M. S., Faridi, S., Abbasi, S., & Gholampour, A. (2022). Long-term trends in ambient air pollutants and the effect of meteorological parameters in Tabriz, Iran. *Urban Climate,**42*, 101119. 10.1016/j.uclim.2022.101119

[CR7] Bilal, M., Mhawish, A., Nichol, J. E., Qiu, Z., Nazeer, M., Ali, M. A., Leeuw, G., Levy, R. C., Wang, Y., Chen, Y., Wang, L., Shi, Y., Bleiweiss, M. P., Mazhar, U., Atique, L., & Ke, S. (2021). Air pollution scenario over Pakistan: Characterization and ranking of extremely polluted cities using long-term concentrations of aerosols and trace gases. *Remote Sensing of Environment, 264*, 112617. 10.1016/j.rse.2021.112617

[CR8] Bilal, M., Nazeer, M., Qiu, Z., Ding, X., & Wei, J. (2018). Global validation of MODIS C6 and C6.1 merged aerosol products over diverse vegetated surfaces. *Remote Sensing,**10*(3), 475. 10.3390/rs10030475

[CR9] Bilal M, Nichol JE, Nazeer M (2016). Validation of Aqua-MODIS C051 and C006 operational aerosol products using AERONET measurements over Pakistan. IEEE Journal of Selected Topics in Applied Earth Observations and Remote Sensing.

[CR10] Bilal M, Nichol JE (2015). Evaluation of MODIS aerosol retrieval algorithms over the Beijing-Tianjin-Hebei region during low to very high pollution events. Journal of Geophysical Research Atmospheres.

[CR11] Boiyo R, Kumar KR, Zhaoa T (2018). Spatial variations and trends in AOD climatology over East Africa during 2002–2016: A comparative study using three satellite data sets. International Journal of Climatology.

[CR12] Che, H., Yang, L., Liu, C., Xia, X., Wang, Y., Wang, H., Wang, H., Lu, X., & Zhang, X. (2019). Long-term validation of MODIS C6 and C6.1 Dark Target aerosol products over China using CARSNET and AERONET. *Chemosphere,**236*, 124268, 10.1016/j.chemosphere.2019.06.238 10.1016/j.chemosphere.2019.06.238 31319316

[CR13] Chen D, Zhang F, Yu C, Jiao A, Xiang Q, Yu Y, Mayvaneh F, Hu K, Ding Z, Zhang Y (2019). Hourly associations between exposure to ambient particulate matter and emergency department visits in an urban population of Shenzhen, China. Atmospheric Environment.

[CR14] Chen, G., Li, Y., Zhou, Y., Shi, C., Guo, Y., & Liu, Y. (2021). The comparison of AOD-based and non-AOD prediction models for daily PM2.5 estimation in Guangdong province, China with poor AOD coverage, *Environmental Research*, *195*, 110735. 10.1016/j.envres.2021.110735 10.1016/j.envres.2021.110735 33460631

[CR15] Chowdhury S, Dey S, Ghosh S, Saud T (2016). Satellite-based estimates of aerosol washout and recovery over India during monsoon. Aerosol and Air Quality Research.

[CR16] Deep A, Pandey CP, Nandan H, Singh N, Yadav G, Joshi PC, Purohit KD, Bhatt SC (2021). Aerosols optical depth and Ångström exponent over different regions in Garhwal Himalaya. India. Environmental Monitoring and Assessment.

[CR17] Elshora, M. (2021). Developing a new IHS-based fusion methodology for GeoEye-1 satellite imagery with the ELSHORA fusion technique. *Journal of Applied Remote Sensing,* 1*5*(4), 046508. 10.1117/1.JRS.15.046508

[CR18] Elshora M (2022). Producing WorldView-2 fused images of superior quality by the novel ELSHORA fusion technique. Applied Geomatics.

[CR19] Falah, S., Mhawish, A., Sorek-Hamer, M., Lyapustin, A. L., Kloog, I., Banerjee, T., Kizel, F., & Broday, D. M. (2021). Impact of environmental attributes on the uncertainty in MAIAC/MODIS AOD retrievals: A comparative analysis. *Atmospheric Environment,* *262*, 118659. 10.1016/j.atmosenv.2021.118659

[CR20] Filonchyk M, Hurynovich V (2020). Validation of MODIS aerosol products with AERONET measurements of different land cover types in areas over Eastern Europe and China. Journal of Geovisualization and Spatial Analysis.

[CR21] Gouda KC, Gogeri I, ThippaReddy AS (2022). Assessment of aerosol optical depth over Indian subcontinent during COVID-19 lockdown (March–May 2020). Environmental Monitoring and Assessment.

[CR22] Gui, K., Che, H., Wang, Y., Xia, X., Holben, B. N., Goloub, P., Agulló, E. C., Yao, W., Zheng, Y., Zhao, H., Li, L., & Zhang, X. (2021). A global-scale analysis of the MISR Level-3 aerosol optical depth (AOD) product: Comparison with multi-platform AOD data sources, *Atmospheric Pollution Research*, *12*(12), 101238. 10.1016/j.apr.2021.101238

[CR23] Hsu NC, Jeong M-J, Bettenhausen C, Sayer AM, Hansell R, Seftor CS, Huang J, Tsay S-C (2013). Enhanced deep blue aerosol retrieval algorithm: The second generation. Journal of Geophysical Research Atmospheres.

[CR24] Ichoku, C., Chu, D. A., Mattoo, S., Kaufman, Y. J., Remer, L. A., Tanré, D., Slutsker, I., & Holben, B. N. (2002). A spatio-temporal approach for global validation and analysis of MODIS aerosol products. *Geophysical Research Letters,**29*(12), MOD1–1-MOD1–4. 10.1029/2001GL013206

[CR25] Jin M, Shepherd JM, King MD (2005). Urban aerosols and their variations with clouds and rainfall: A case study for New York and Houston. Journal of Geophysical Research Atmospheres.

[CR26] Kang, N., Deng, F., Khan, R., Kumar, K. R., Hu, K., Yu, X., Wang, X., & Devi, N. S. M. P. L. (2020). Temporal variations of PM concentrations, and its association with AOD and meteorology observed in Nanjing during the autumn and winter seasons of 2014–2017. *Journal of Atmospheric and Solar-Terrestrial Physics,**203*, 105273. 10.1016/j.jastp.2020.105273

[CR27] Kaufman, Y. J., Wald, A. E., Remer, L. A., Gao, B-C., Li R-R., & Flynn, L. (1997). The MODIS 2.1-/spl mu/m channel-correlation with visible reflectance for use in remote sensing of aerosol. *IEEE Transactions on Geoscience and Remote Sensing,**35*(5), 1286–1298. 10.1109/36.628795

[CR28] Kayes I, Shahriar SA, Hasan K, Akhter M, Kabir MM, Salam MA (2019). The relationships between meteorological parameters and air pollutants in an urban environment. Global Journal of Environmental Science and Management.

[CR29] Kim, S.-M., Koo, J.-H., Lee, H., Mok, J., Choi, M., Go, S., Lee, S., Cho, Y., Hong, J., Seo, S., Lee, J., Hong, J.-W., & Kim, J. (2021). Comparison of PM2.5 in Seoul, Korea estimated from the various ground-based and satellite AOD. *Applied Sciences,**11*(22), 10755. 10.3390/app112210755

[CR30] Lalitaporn P, Mekaumnuaychai T (2020). Satellite measurements of aerosol optical depth and carbon monoxide and comparison with ground data. Environmental Monitoring and Assessment.

[CR31] Levy RC, Remer LA, Kleidman RG, Mattoo S, Ichoku C, Kahn R, Eck TF (2010). Global evaluation of the Collection 5 MODIS dark-target aerosol products over land. Atmospheric Chemistry and Physics.

[CR32] Lipponen A, Mielonen T, Pitkänen MRA, Levy RC, Sawyer VR, Romakkaniemi S, Kolehmainen V, Arola A (2018). Bayesian aerosol retrieval algorithm for MODIS AOD retrieval over land. Atmospheric Measurement Techniques.

[CR33] Makokha JW, Odhiambo JO, Godfrey JS (2017). Trend analysis of aerosol optical depth and Ångström exponent anomaly over East Africa. Atmospheric and Climate Sciences.

[CR34] Nichol JE, Bilal M, Ali MA, Qiu Z (2020). Air pollution scenario over China during COVID-19. Remote Sensing.

[CR35] Osgouei, P. E., Roberts, G., Kaya, S., Bilal, M., Dash, J., & Sertel, E. (2022). Evaluation and comparison of MODIS and VIIRS aerosol optical depth (AOD) products over regions in the Eastern Mediterranean and the Black Sea. *Atmospheric Environment,**268*, 118784. 10.1016/j.atmosenv.2021.118784

[CR36] Ramachandran S, Kedia S, Srivastava R (2012). Aerosol optical depth trends over different regions of India. Atmospheric Environment.

[CR37] Remer LA, Kaufman YJ, Tanré D, Mattoo S, Chu DA, Martins JV, Li R-R, Ichoku C, Levy RC, Kleidman RG, Eck TF, Vermote E, Holben BN (2005). The MODIS aerosol algorithm, products, and validation. Journal of the Atmospheric Sciences.

[CR38] Su, Y., Xie, Y., Tao, Z., Hu, Q., Yu, T., & Gu, X. (2021). Validation and inter-comparison of MODIS and VIIRS aerosol optical depth products against data from multiple observation networks over East China. *Atmospheric Environment, 247*, 118205. 10.1016/j.atmosenv.2021.118205

[CR39] Tariq, S., Nawaz, H., Ul-Haq, Z., & Mehmood, U. (2021). Investigating the relationship of aerosols with enhanced vegetation index and meteorological parameters over Pakistan. *Atmospheric Pollution Research,**12*(6), 101080, 10.1016/j.apr.2021.101080

[CR40] Tian, X., & Gao, Z. (2019). Validation and accuracy assessment of MODIS C6.1 aerosol products over the heavy aerosol loading area. *Atmosphere,**10*(9), 548. 10.3390/atmos10090548

[CR41] Wang D, Zhang F, Yang S, Xia N, Ariken M (2020). Exploring the spatial-temporal characteristics of the aerosol optical depth (AOD) in Central Asia based on the moderate resolution imaging spectroradiometer (MODIS). Environmental Monitoring and Assessment.

[CR42] Wang Q, Sun L, Wei J, Yang Y, Li R, Liu Q, Chen L (2017). Validation and accuracy analysis of global MODIS aerosol products over land. Atmosphere.

[CR43] Wang W, Mao F, Pan Z, Du L, Gong W (2017). Validation of VIIRS AOD through a comparison with a sun photometer and MODIS AODs over Wuhan. Remote Sensing.

[CR44] Wei J, Li Z, Peng Y, Sun L (2019). MODIS Collection 6.1 aerosol optical depth products over land and ocean: Validation and comparison. Atmospheric Environment.

[CR45] Wei J, Li Z, Sun L, Peng Y, Wang L (2019). Improved merge schemes for MODIS Collection 6.1 Dark Target and Deep Blue combined aerosol products. Atmospheric Environment.

[CR46] Wei J, Peng Y, Guo J, Sun L (2019). Performance of MODIS Collection 6.1 Level 3 aerosol products in spatial-temporal variations over land. Atmospheric Environment.

